# Satisfaction of search: evidence of premature termination in a naturalistic image inspection task

**DOI:** 10.1186/s41235-026-00750-w

**Published:** 2026-08-28

**Authors:** Andrew Hollingworth, Zexuan Niu, Claudia Mello-Thoms, Cathleen M. Moore

**Affiliations:** 1 Department of Psychological and Brain Sciences, The University of Iowa, Iowa City, IA 52242 USA; 2 Department of Radiology, The University of Iowa, Iowa City, IA 52242 USA

**Keywords:** Medical image analysis, Radiology, Visual search, Satisfaction of search

## Abstract

In radiology and in traditional visual search tasks, the detection of a first target in an image tends to impair the detection of additional targets, a phenomenon termed *satisfaction of search* (SOS) or *subsequent search misses*. The original explanation for SOS focused on early termination of inspection after having found a first target. Tests of this hypothesis have yielded mixed results, however. Here, we developed a novel task to examine the potential role of early termination in SOS. In our “Ghosts in the Forest” task, the visual search backgrounds were contour-dense natural scene images, and targets were semi-transparent face images drawn from two emotion categories: happy and angry. Targets were either high salience (HS) or low salience (LS), and scenes contained either one HS target, one LS target, or both an HS target and an LS target. First, we observed a robust SOS effect for the detection of LS targets, both in a sample of naïve observers (Experiment 1) and in a sample of radiologists (Experiment 2). Second, we observed an early termination effect following HS target detection. Third, the time spent searching after detecting an HS target reliably predicted the magnitude of the SOS effect at an individual participant level: SOS was most pronounced for those participants who terminated search earlier. Together, the results indicate that early termination of image inspection is a substantial cause of SOS in a task that embodies some of the core properties of medical image analysis.

## Significance

False negatives are common in the radiological diagnosis of cancer. A significant cause of these errors is reduced detection rate after having detected a first abnormality in an image. Here, we developed an analog of medical image analysis to examine the role of early search termination in such errors. Second-target misses were associated with early termination, with potential applications for optimizing medical image reading.

## Introduction

In the radiological detection of cancer, false negatives are common. For example, nearly 320,000 new breast cancer cases are expected to be diagnosed in the USA in 2025, accounting for more than 30% of anticipated new cancer cases (Siegel et al., 2025). Yet, despite the large number of identified cases, and despite substantial improvements in screening technology, the false-negative rate has remained essentially unchanged at 10 to 20% since the early 1990s (Bird et al., 1992; Lee et al., 2023). These persistently high miss rates suggest that human limitations in perception and decision making contribute substantially to their occurrence (Rich et al., 2008).

One potential source of human error in the radiological detection of cancer is “satisfaction of search” (SOS). SOS refers to the phenomenon that after having found one abnormality, the miss rate for additional abnormalities in the same case is substantially elevated (Smith, 1967; Tuddenham, 1962). SOS errors can be especially problematic if an initially detected abnormality is a false positive, and the subsequently missed abnormality is a malignant lesion (Mello-Thoms et al., 2005). Importantly, experimental studies have confirmed that abnormalities that are missed through SOS are detected at relatively high rates when they are the only abnormality (Berbaum et al., 1990). Thus, SOS errors cannot be attributed to the overall fidelity of an image or to low signal-to-noise ratio of the abnormality. Instead, SOS errors seem to reflect human error in perception and/or decision making (Berbaum et al., 2010).

As the name of the phenomenon suggests, SOS was originally thought to be caused by premature termination of the inspection of a case after an initial detection of an abnormality (Smith, 1967; Tuddenham, 1962). The idea is that because the goal of medical interpretation of images is to distinguish images with abnormalities from those without abnormalities, once an abnormality is detected, the goal is satisfied, and the reading can be terminated. Experimental studies testing the premature-termination hypothesis, however, have yielded mixed results. For example, Berbaum et al. (1991) found that the time taken by radiologists to read chest radiographs was unaffected by whether there was a single abnormality or two abnormalities, even though there was a reliable SOS effect. They used chest radiographs that included an abnormality, and for half of the images they superimposed a second artificial abnormality (a nodule) that was more salient than the native abnormality. In addition to the hit and false-positive rates for the two types of images, they measured response time for various parts of the readings. These included the time that the salient artificial nodule was detected, the time that the native abnormality was detected, and the time that the reading of the case was terminated. An SOS effect was observed in that the false-negative rate for the native abnormalities was higher for images that had the additional nodule than for those that did not. There was, however, no significant difference in the total inspection time (i.e., the time to case termination) for images with an additional nodule than without one. There was also no significant difference in the time taken to report native abnormalities that were reported in images with additional nodules than those without them. These results suggested that the SOS effect was not necessarily attributable to premature termination of inspection as originally suspected.

In another study, Berbaum et al. (1998) measured gaze position while radiologists read cases. They found that gaze dwell times on native abnormalities were the same for radiographs that included additional salient abnormalities as for those that did not. Although those results seem to challenge the premature-termination hypothesis, a later study using chest computed tomography (CT) scans yielded results that were consistent with premature termination (Berbaum et al., 2013). Inspection times were analyzed for cases in which there was a native abnormality, and an additional salient nodule was superimposed for half of the cases. Unsurprisingly, the range of slices that included the nodule were inspected for a longer period (9 s longer, on average) than those same slices that did not have the superimposed nodule. In addition, however, the remaining slices were inspected for a *shorter* period (12 s shorter, on average) for cases that included the artificial nodule compared to those that did not. Therefore, although there was no significant difference in total inspection time, post-detection slices were inspected for significantly less time when the nodule was present than when it was not. The participants in this study were mainly radiology residents and fellows, rather than more experienced radiologists, so it is possible that this sample contributed to the pattern of observed results. Finally, in a study that was similar to the Berbaum et al. (1991) study but with a deadline of 30 s for each case, radiographs with two abnormalities were scrutinized for a shorter period of time than were radiographs with only one abnormality (Samuel et al., 1995).

Given the mixed results concerning the possibility of premature termination, two additional (and not mutually exclusive) alternative explanations have been suggested. One appeals to perceptual biases generated by first-target detection. According to this *perceptual-set hypothesis*, finding an abnormality of one type can bias readers to look for similar abnormalities, thereby making the reader vulnerable to missing other types of abnormality (e.g., Berbaum et al., 1990, 1991). In mechanistic terms, the perceptual-set hypothesis proposes that detecting a target causes the search template to be tuned toward the initial abnormality, thereby making it less sensitive to abnormalities that do not closely resemble it. Another alternative explanation of SOS errors appeals to the limited availability of processing resources. According to the *resource-depletion hypothesis*, when an initial abnormality is detected, processing resources are dedicated to understanding it, thereby reducing the resources available for detecting a second abnormality (Berbaum et al., 1991; Cain & Mitroff, 2013; Cain et al., 2013).

Given the complexity of medical images and the challenges of conducting experiments with a limited population of trained radiologists, researchers have tested these alternative explanations of SOS errors using basic visual search tasks, which are relatively easy to control and can be performed without extensive training (e.g., Adamo et al., 2015, 2017, 2019; Cain & Mitroff, 2013; Fleck et al., 2010). In Fleck et al. (2010), for example, young adult participants with no training in radiology searched displays of multiple discrete stimuli for targets (Ts) among distractors (Ls). Trials included 0, 1, or 2 targets. Some targets were more salient than others, such that when there were two targets, the more salient target was usually found first. The measure of SOS was the difference between low-salience target detection percentage when the low-salience target was the only target in the array versus when a high-salience target was also present in the array. In this context, the atheoretical term *subsequent search misses* (SSM) was preferred, because the goal was to test alternative sources of the errors (Adamo et al., 2021; Cain et al., 2013). A recent review summarized this literature as showing substantial support for both the perceptual-set and the resource-depletion hypotheses, but only mixed support for the premature-termination hypothesis (Adamo et al., 2021). That review concluded with the introduction of an attention-based explanation—the *attentional template theory*—which is an effort to unify the findings. Premature termination is not a significant contributor to SOS errors in that theory. A similar conclusion was drawn in the medical image analysis literature by Kundel and Nodine (2018), who concluded that SOS was driven primarily by perceptual-level factors (i.e., recognition failure) rather than by scanning or visual search processes, such as early termination.

The goal of the current study was to investigate the source of SOS errors using a task that recovers several key features of the radiology task while retaining the benefits of the basic visual search tasks used more recently to understand the specific mechanistic causes of SOS. Reading medical images for signs of abnormality introduces several specific demands. First, medical images tend to be visually complex and more complex than traditional visual search displays, even when the visual search displays are superimposed on a cloudy noise background intended to capture some of the quality of radiographs (Fleck et al., 2010). Second, medical image reading is an image-based task, in which stimuli do not necessarily consist of discrete elements to which search can be guided. This contrasts with traditional visual search, which is typically an item-based task (c.f. Hulleman & Olivers, 2017), in which discrete elements that are clearly segmented from the background are individually classified as targets or distractors based on well-defined, prespecified criteria (e.g., T vs. L). The challenges of reading medical images may therefore be more similar to the challenges of detecting figures in camouflage than the challenges of attentional guidance within arrays of items. Finally, radiologists face substantial pressures on their time, as they are often expected to complete a certain number of readings within a fixed period (Alexander et al., 2022). Time pressure may be important to observing early termination and SOS (Samuel et al., 1995), as radiologists may choose to terminate readings with a single detected abnormality relatively early given the demand to allocate time to other cases. For these reasons, we sought to develop a task that (1) has the advantages of the basic visual search tasks which have been productive in identifying SOS causes (tight experimental control and the option of testing participants without specialized expertise) and (2) incorporates several task-specific features of visual search during medical image reading, including: visually dense, complex images; an image-based target detection task; and a deadline to generate pressure on participants’ allocation of time to each image.

**Fig. 1:**
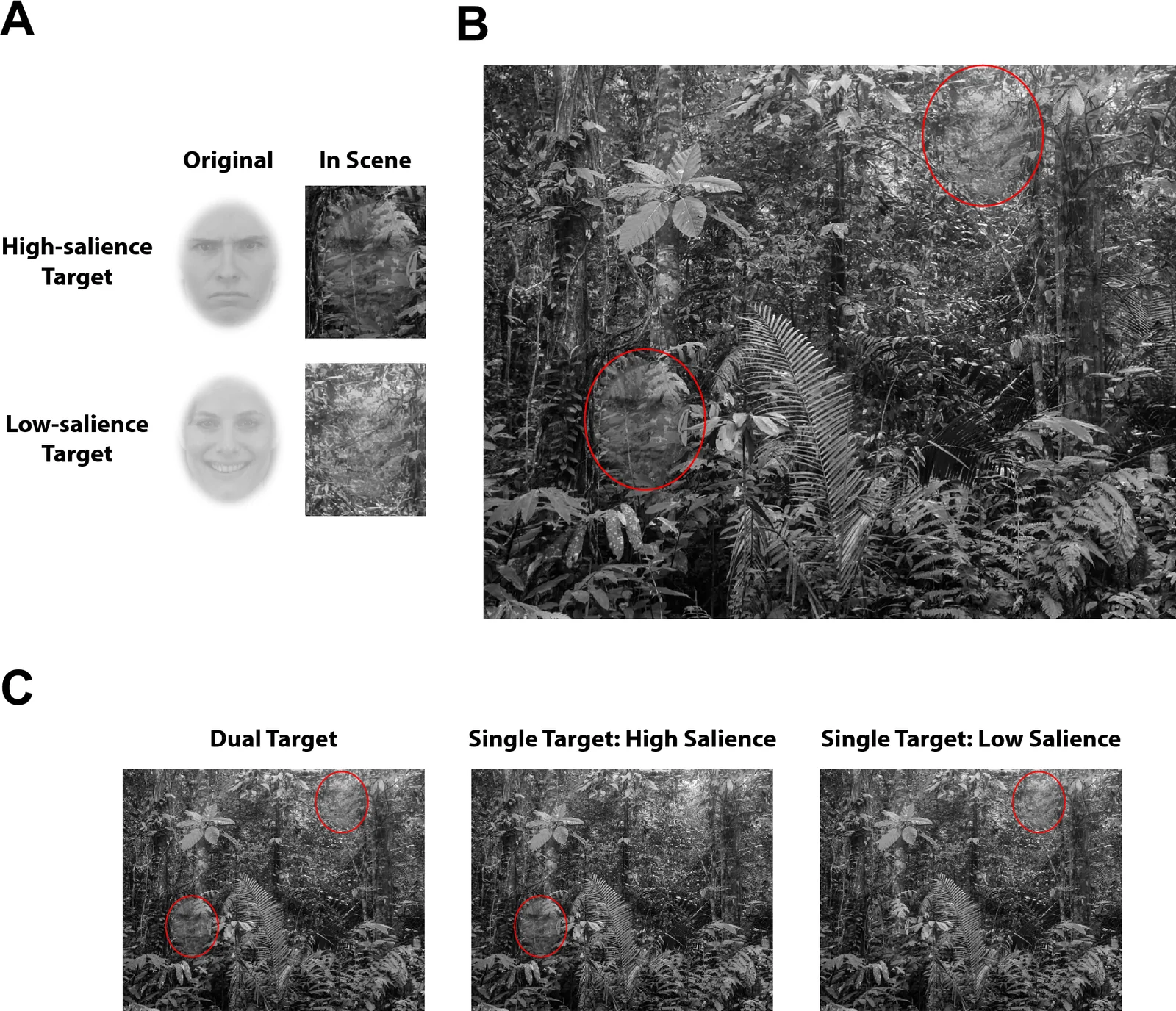
Stimulus design. Happy and angry faces (**A**) were presented within contour-dense images of forest and jungle scenes (**B**). Each face was camouflaged within the scene by setting the opacity level of the face to 40%, with a Gaussian opacity transition at the edges of the crop. A visual similarity algorithm (between the face and local regions of the scene) was used to generate one embedded face target of relatively high visual salience (HS) and one of relatively low visual salience (LS). Each scene item contained one HS and one LS target (**C**). Two additional versions of each scene were created by removing one of the two targets. The three scene versions in this “matched-triplet” design were counterbalanced across participant subgroups. Note that the red ovals indicate the locations of the face targets and were not present in the experimental stimuli

**Fig. 2:**
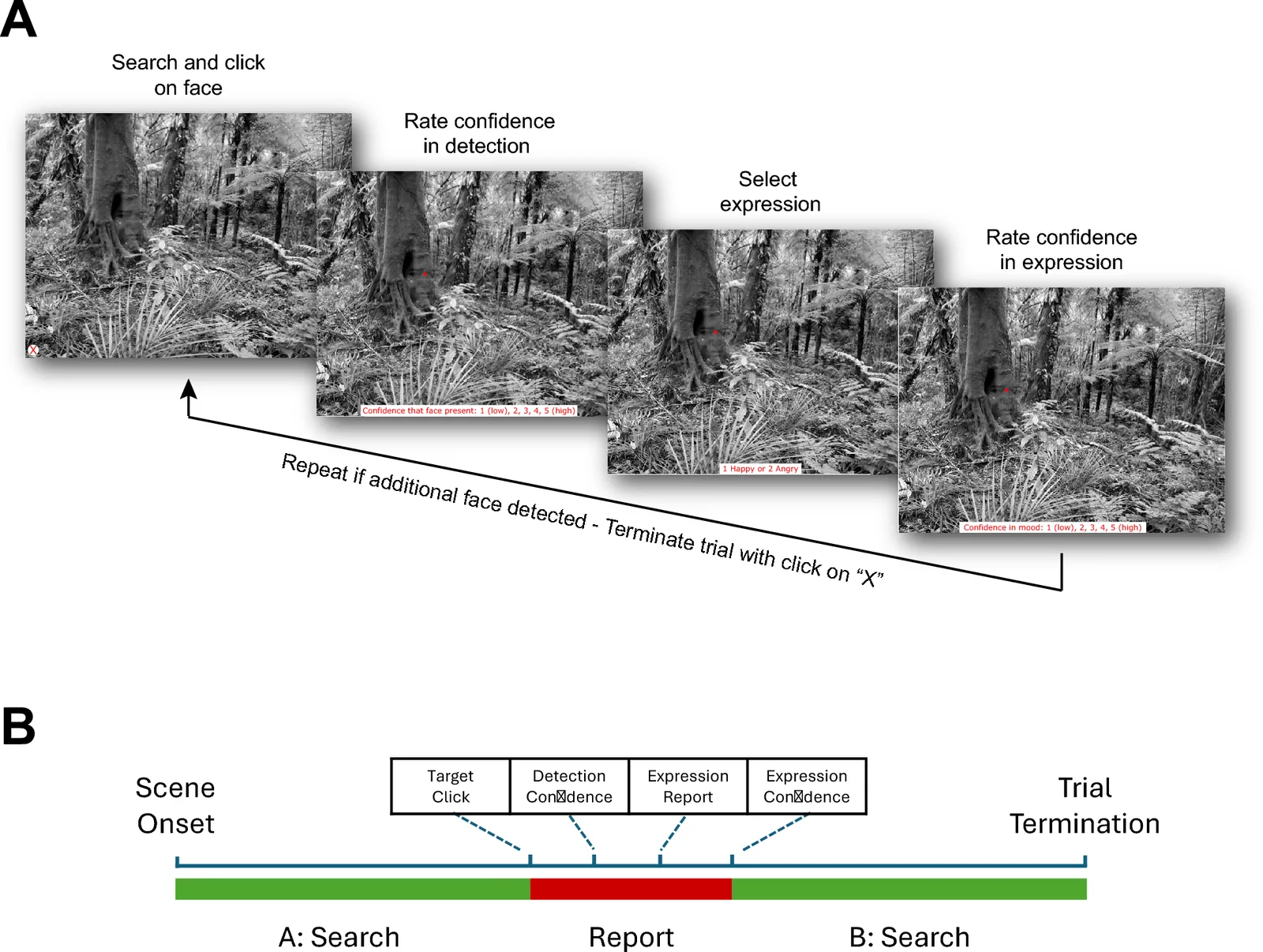
**A** Sequence of events in a trial. Participants searched for face images. When a face was detected, the participant clicked on it. This triggered a series of response screens: (1) confidence in the presence of a face, (2) expression selection, and (3) confidence in expression selection. Participants then resumed search and repeated the detection/report processes until they were confident that all faces had been found, clicking the “X” to terminate the trial. **B** Illustration of the subtraction method used to calculate search time. Total search time was the total trial time minus any periods of target report (depicted in red). Post-target search time was the time devoted to search after having finished report for the first detected target (search period B)

The stimuli and task developed and used in the current study are illustrated in Figs. 1 and 2. Images of contour-dense natural scenes were used as backgrounds, mimicking some of the image properties of dense breast tissue. Zero, one, or two target stimuli—analogous to medical cases with zero, one, or two abnormalities—were superimposed onto the backgrounds. The targets were semi-transparent faces drawn from two different emotion categories: happy and angry. We employed a local image-based similarity algorithm to manipulate the salience of each face within the scene. Low-salience (LS) targets were placed in a local region of high image-based similarity, making them relatively difficult to detect, and high-salience (HS) targets were placed in a local region of relatively low image-based similarity, making them relatively easy to detect. In addition, targets were smoothly integrated with the background so there was no visible discontinuity that could be used to parse the display into a set of discrete candidate items.

For each scene, we created a base image with two targets, one HS and one LS. Two single-target images were then created by removing one of the two targets. Thus, single-target images were identical to the corresponding dual-target image except for the presence of the second target. This ensured strong control over all non-manipulated stimulus properties (Adamo et al., 2019). The three versions of each scene were then counterbalanced across three participant groups. SOS was operationalized, generally, as a lower probability of target detection for targets in dual-target images than for targets in single-target images. More specifically, however, given that HS targets were expected to be detected at a very high rate, SOS was measured as a higher detection rate for LS targets when they appeared alone compared with when they appeared in the same scene with an HS target.

We tested two groups of participants in this task: a relatively large group of naïve participants with no radiological experience and a smaller group of working radiologists. Each was required to complete the experiment in an average of 30 s per image (Samuel et al., 1995). Both groups showed reliable SOS, and analyses of inspection times yielded evidence that premature termination of search was a substantial cause of the effect, both for the naïve participants and for the radiologists.

## Experiment 1

### Method

#### Participants

We aimed to include 60 naïve participants drawn from the general population (i.e., with no specific expertise in radiology). The choice of N was based on an online pilot study similar to Experiment 1 (*N* = 36). For the key contrast (within-participant difference between detection rates for LS faces presented with an HS face in the same image or without an HS face in the same image), we observed an effect on detection rate of *d*_*z*_ = 0.46, indicating that an *N* of 40 would be sufficient to ensure 80% power at *α* = .05, calculated using G*Power. Conservatively, we chose an *N* of 60. Participants were recruited from the Prolific recruitment system (www.Prolific.com). Inclusion was limited to native speakers of English between the ages of 18 and 30 located in the USA. In addition, participants were required to complete the experiment on a desktop computer. They were paid $8.00 for the approximately 40 min required to complete the experiment. One participant was replaced due to a period of inactivity during the session that lasted more than one hour. For the final set of 60 participants (32 female, 28 male; mean age = 25.7 years), 20 were included in each of the three counterbalancing subgroups.

#### Apparatus

The experiment was implemented in OpenSesame software and hosted on a JATOS server maintained by the University of Iowa. Participants completed the experiment using their own desktop computer. Given variation in monitor size and viewing distance, we report stimulus size in pixels.

#### Stimuli

The stimulus set consisted of 36 experimental scenes and 24 filler scenes. All scenes were chosen to be contour-dense images of forests or jungles (See Figs. 1 and 2 for examples) at a resolution of 1024 × 768 pixels. Images were acquired from the Adobe Stock database. Face stimuli were obtained from the Karolinska Directed Emotional Faces (KDEF) database (Lundqvist et al., 1998). The entire face set consisted of 120 images: 30 female and 30 male faces, each in two expressions (happy and angry). The face images had an original resolution of 562 × 762 pixels. They were converted to grayscale and cropped using an oval mask that isolated the central part of the face. Each face was set to an opacity level of 40%. In addition, a 15-pixel, Gaussian opacity transition (from 40 to 0%) was applied to the border of the crop to eliminate hard edges when placed in the scenes. For each of the 120 images, four versions were created at four different sizes: 77 × 100 pixels, 98 × 128 pixels, 119 × 155 pixels, and 140 × 182 pixels.

To create the search stimuli, two faces from the set of 120 were selected to be placed in each of the 36 experimental scene images. This allowed for identity repetitions, but no specific combination of identity and expression was repeated. The size of each picture was randomly selected. A local visual similarity algorithm was used to identify locations in each scene where one face would have relatively low similarity with the local background (HS target) and one would have relatively high similarity with the local background (LS target). Specifically, we used the TM_CCORR template-similarity algorithm in Python OpenCV (Bradski, 2000), which summed the products of pixel intensities between the face image and an equivalently sized region of the scene. This was applied to every possible location within the scene images, and the similarity scores at each location were rank ordered. LS target locations were chosen randomly from the top 25% of similarity scores. HS target locations were chosen randomly from locations ranked in the 40–50% range of similarity scores. Six versions of each scene were created with the two faces at different locations that met similarity criteria. From these six, one version was hand selected to ensure that the two faces were sufficiently separated spatially and that they did not appear in the very bottom region of the scene, where response options would be displayed. Twelve of the scenes contained one happy face and one angry face (six in which the happy face was HS and the angry face LS; six with the reverse assignment), 12 contained two happy faces, and 12 contained two angry faces. Two facial expression categories were used to reflect the fact that abnormalities in radiological images may come from different categories (e.g., benign and cancerous lesions in a mammogram).

For each of the experimental scenes, we created a base image with both targets: one HS and one LS. Two single-target images were then created by removing one of the two targets. Thus, single-target images were identical to the corresponding dual-target image except for the presence of the second target. This ensured control over all non-manipulated stimulus properties (Adamo et al., 2019). The three versions of each scene were then counterbalanced across three participant subgroups so that each participant saw each scene item only once.

There were also 24 filler scenes. Twelve of the filler scenes were created with only one HS face (six happy and six angry). The final 12 filler scenes had no targets. The filler items were the same across the three participant subgroups.

#### Procedure

Upon clicking the link to initiate the experiment, the participant provided informed consent and received instructions. The instructions specified that the task was to find “ghosts in the forest.” Participants were told that each scene would contain either zero, one, or two “ghostly” faces. Their task was to find all the faces in each image. They should use the mouse to click on each face and then indicate whether it was happy or angry. They were also instructed that during the main session, they should strive to complete all 60 scenes within 30 min.

The trial events are displayed in Fig. 2. First, the search scene was displayed until the participant either (1) clicked on the scene to indicate detection of a face or (2) clicked on the red “X” in a lower-left corner to indicate that they had finished searching. When a mouse click was detected on the scene, a red dot was presented at the clicked location, and a response field was added to the bottom of the screen asking the participant to rate confidence that a face was present at that location. The participant used the keyboard to enter a response between 1 (low confidence) and 5 (high confidence). Next, a second response field asked the participant to indicate the facial expression, entering “1” for happy or “2” for angry. Finally, a third response field collected confidence in the expression judgment on the 1–5 scale. Following this final response, the scene returned to the base version. Participants continued this cycle of search and report until they were satisfied that they had found all the faces in the image, clicking the red “X” to terminate the trial. Clicks on the image were coded as correct detections if the click location fell within a rectangular region containing a face, with the regions defined by the boundaries of each face image.

Before completing the main session, participants completed a practice session of eight trials: two dual-target scenes, five single-target scenes (three LS and two HS), and one no-target scene. In the practice session, the participants were provided feedback. After trial completion, they saw the scene image with the target(s) outlined in white. After pressing the spacebar to continue, they viewed a text screen that listed (1) the number of faces in the preceding scene, (2) which faces had and had not been detected, and (3) whether the facial expressions were reported correctly. In addition, if the participant clicked on a scene location that did not contain a face, the following text was displayed: “You clicked on at least one location that DIDN'T contain a ghost. The forest gods are displeased.”

The main session consisted of 60 trials (36 experimental scenes and 24 filler scenes), with trial order randomly determined. No direct feedback was provided in the main session. Following trial completion, a message was displayed that indicated how many of the 60 scenes had been completed, with countdown timer indicating the number of minutes remaining (out of a total of 30 min). No participants exceeded the allotted time. Participants pressed the spacebar to proceed to the next scene.

#### Transparency and openness

We report how we determined our sample size, all data exclusions (if any), all manipulations, and all measures in the study. The study was not pre-registered.

## Results and discussion

We first report detection rates to assess the presence and magnitude of the predicted SOS effect. We then assess search termination times to examine whether the SOS effect can be attributed to early termination of search after having found a first target in dual-target scenes. Finally, we report whether individual differences in termination times predict detection rates for LS targets and for the difference between LS detection rates in single- versus dual-target scenes (i.e., the SOS effect). The rates and response times for all possible trial outcomes are reported in Table 1.

**Table 1:** Experiment 1 results

	Percentage of trials	Search time before detection (s)^a^		Total time before detection (s)^b^		Total search time (s)^c^	Total trial time (s)
		First target	Second target	First target	Second target		
2 Target							
Both detect: HS first	41.11	2.32	7.67	2.32	11.50	11.97	20.73
Both detect: LS first	5.56	*	*	*	*	*	*
HS detect only	48.33	3.38	NA	3.39	NA	12.81	16.88
LS detect only	1.39	NA	*	NA	*	*	*
Both miss	3.61	NA	NA	NA	NA	*	*
1 Target HS							
Detect	95.00	2.88	NA	2.91	NA	11.41	15.42
Miss	5.00	NA	NA	NA	NA	*	*
1 Target LS							
Detect	58.33	7.62	NA	7.65	NA	14.41	19.46
Miss	41.67	NA	NA	NA	NA	16.49	16.90
0 Target filler		NA	NA	NA	NA	17.17	17.52

^a^Time from trial onset to target detection, subtracting report time for any detections or false alarms that occurred earlier in the trial

^b^Time from trial onset to target detection, including report time for any detections or false alarms that occurred earlier in the trial

^c^Total time devoted to search: total trial time subtracting report time for detections and false alarms

*: More than 25% of participants had no observations, precluding a stable estimate of the mean

### Detection rates, detection times, and false alarm rates

**Fig. 3:**
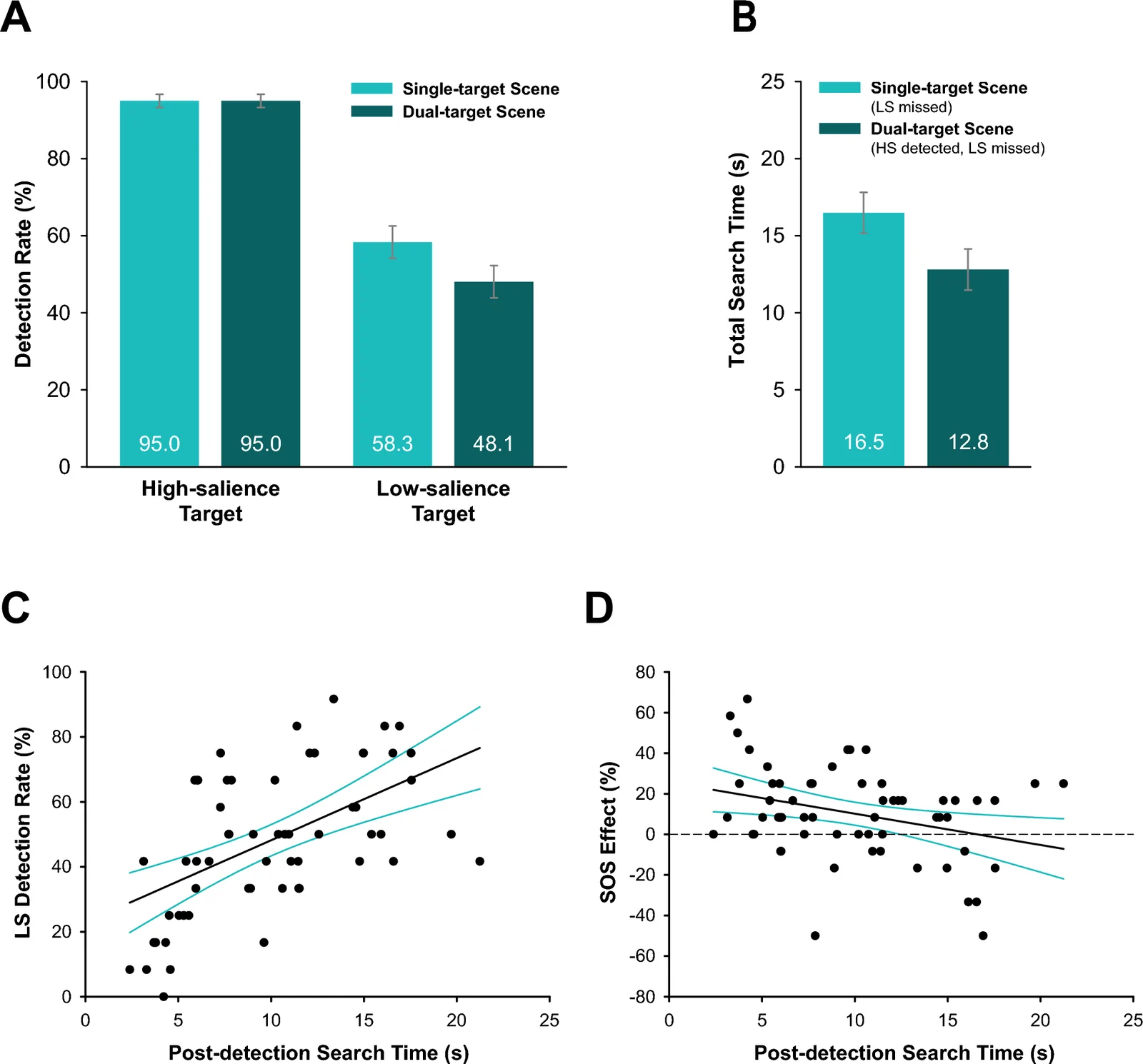
Experiment 1 results. **A** Detection rates as a function of target salience and the number of targets in a scene. Error bars are within-participant 95% confidence intervals based on the error term of each of the grouped contrasts (Morey, 2008). **B** Time devoted to search for (1) single-target scenes in which the LS target was missed and for (2) dual-target scenes in which the HS was detected and the LS target missed. Error bars are within-participant 95% confidence intervals. **C** Relation between an individual’s post-detection search time (time devoted to search after having detected an HS target in a single-target scene) and detection rate for LS targets in dual-target scenes. **D** Relation between post-detection search time and an individual’s SOS rate (detection rate for LS target in single-target scenes minus detection rate for LS targets in dual-target scenes). Blue-green lines are 95% confidence bounds

The primary analyses concerned detection rates over the 36 experimental scenes, as illustrated in Fig. 3A. The detection rate data were entered into 2 (HS/LS face target) × 2 (single-/dual-target scene) repeated measures ANOVA. First, there was a reliable main effect of target salience, *F*(1,59) = 405.0, *p* < .001, $\eta_{p}^{2} = 0.873,$ with higher detection rate for HS targets than for LS targets. Second, there was a reliable main effect of single-/dual-target scene, *F*(1,59) = 9.34, *p* = .003, $\eta_{p}^{2} = 0.137,$ with higher detection rate for targets that appeared in single-target scenes than in dual-target scenes. Finally, there was a reliable interaction, *F*(1,59) = 11.59, *p* = .001, $\eta_{p}^{2} = 0.164,$ driven by the fact that the effect of the number of targets in a scene was limited to LS targets. We then conducted two planned contrasts. As expected, HS targets were detected at near-ceiling levels, with a mean detection rate of 95.0%, both when the HS target appeared in isolation and when it appeared in the presence of an LS target in dual-target scenes. The key contrast assessing SOS was the detection rate for LS targets as a function of whether the LS target appeared in isolation or in the presence of an HS target. These reliably differed, *t*(59) = 3.47, *p* < .001, *d*_*z*_ = 0.49, with higher detection rate when an LS target appeared in isolation (*M* = 58.3%) than when an LS target appeared with an HS target (*M* = 48.1%). Note again that the LS targets were physically identical across conditions, with the only difference being the presence of an HS target at a different location within the scene.

We also calculated SOS by considering only those dual-target trials where an HS face was the first or only target detected. This isolates the situation of primary interest: detection probability for LS targets after an HS target had been found. For dual-target trials, there are five possible outcomes. The grand participant means for the percentage of trials in each of the five outcomes were: (1) Both targets detected and HS detected before LS (41.1%), (2) both targets detected and LS detected before HS (5.6%), (3) HS detected and LS missed (48.3%), (4) LS detected and HS missed (1.4%), and 5) both missed (3.6%). The contingent analysis was restricted to outcomes 1 and 3, that is, trials on which the HS target was the first or only detected target. Under this contingency, the mean LS detection rate in dual-target scenes was 46.2%.

To evaluate whether this reflected a genuine SOS effect, we compared the observed contingent LS detection rate against the unbiased null expectation proposed by Becker et al. (2020). As Becker et al. noted, the traditional contingent analysis is inherently biased because it excludes all dual-target trials on which the LS target was detected before the HS target, thereby selectively removing relatively easy LS detections from the dual-target estimate while retaining those trials in the single-target estimate. This produces an artificial reduction in contingent LS detection performance even in the absence of any true dual-target deficit.

To correct for this bias, Becker et al. derived the expected contingent LS detection rate under the null hypothesis that detection of the HS target does not impair LS detection. This expected value is:

1 $$P\_\mathrm{e}\mathrm{x}\mathrm{p}\mathrm{e}\mathrm{c}\mathrm{t}\mathrm{e}\mathrm{d}=\frac{P\left(\mathrm{L}\mathrm{S}\right) * P(\mathrm{H}\mathrm{S} \mathrm{t}\mathrm{h}\mathrm{e}\mathrm{n} \mathrm{L}\mathrm{S})}{P\left(\mathrm{L}\mathrm{S}\right) * P\left(\mathrm{H}\mathrm{S} \mathrm{t}\mathrm{h}\mathrm{e}\mathrm{n} \mathrm{L}\mathrm{S}\right) +1 - P(\mathrm{L}\mathrm{S})}$$

where *P*(LS) is the observed LS detection rate in single-target scenes and *P*(HS then LS) is the proportion of dual-target detections on which the HS target was detected before the LS target. Applying this correction yielded an expected contingent LS detection rate of 55.4%.

The observed contingent LS detection rate in dual-target scenes (46.2%) remained significantly lower than this corrected expectation, *t*(59) = 3.08, *p* = .003, *d*_z_ = 0.40. Thus, the SOS effect remained significant even after correcting for the selection bias inherent in traditional contingent dual-target analyses.

Consistent with the difference in detection rates for HS and LS targets, detection times reliably differed as a function of salience. For correct detections over the 36 experimental scenes, we calculated mean time from the onset of the search array to the click on a face target, subtracting the time taken to respond to any targets that were detected earlier within the trial. One participant with no LS detections in dual-target scenes was eliminated from the analysis and from subsequent analyses of confidence and expression report. For the remaining 59 participants, this measure was analyzed as a function of HS/LS target and single-/dual-target scene (Means: HS single = 2.90 s, HS dual = 3.08 s, LS single = 7.65 s, LS dual = 7.09 s). There was a main effect of salience, *F*(1,58) = 168.9, $p\, < \,.001,$ $\eta_{p}^{2} = 0.744,$ but no effect of single-/dual-target scene, *F*(1,58) = 0.567, *p* = .455, $\eta_{p}^{2} = 0.010,$ and no interaction, *F*(1,58) = 2.16, *p* = .147, $\eta_{p}^{2} = 0.036.$

Confidence in the presence of a detected target (scale of 1–5) was analyzed as a function of HS/LS and single-/dual-target scene (Means: HS single = 4.74, HS dual = 4.75, LS single = 3.35, LS dual = 3.47). There was a main effect of salience, *F*(1,58) = 216.7, *p* < .001, $\eta_{p}^{2} = 0.789,$ but no effect of single-/dual-target scene, *F*(1,58) = 0.98, *p* = .326, $\eta_{p}^{2} = 0.017,$ and no interaction, *F*(1,58) = 0.712, *p* = .402, $\eta_{p}^{2} = 0.012$.

We also examined the accuracy of expression report (Means: HS single = 90.3%, HS dual = 92.6%, LS single = 73.8%, LS dual = 71.6%). There was a main effect of salience, *F*(1,58) = 73.32, *p* < .001, $\eta_{p}^{2} = 0.558,$ but no effect of single-/dual-target scene, *F*(1,58) = 0.0003, *p* = .985, $\eta_{p}^{2} = 0.00,$ and no interaction, *F*(1,58) = 1.70, *p* = .197, $\eta_{p}^{2} = 0.029.$

For expression confidence (Means: HS single = 4.24, HS dual = 4.31, LS single = 2.54, LS dual = 2.86), there was a main effect of salience, *F*(1,58) = 325.0, *p* < .001, $\eta_{p}^{2} = 0.849,$ a reliable main effect of single-/dual-target scene, *F*(1,58) = 10.09, *p* = .002, $\eta_{p}^{2} = 0.148,$ and a reliable interaction, *F*(1,58) = 4.08, *p* = .048, $\eta_{p}^{2} = 0.066.$ These confidence ratings for expression report are difficult to interpret, however, given differences in detection rates among conditions.

False alarm rates were low. For the 36 experimental stimuli, participants clicked on a location that did not contain a face on an average of 4.58% (SD = 7.23%) of trials for single-target scenes and 3.19% (SD = 6.15%) of trials for dual-target scenes.

In addition, we report detection rates as a function of the factors that were not of direct experimental interest. Overall, the detection rate for angry faces in the 36 experimental scenes was 77.8%, and the detection rate for happy faces was 70.5%. For the four target sizes, the detection rates were 70.8%, 76.4%, 71.7%, and 79.2%, from smallest size to largest size, respectively. For the 12 single-target filler scenes (all HS), the mean detection rate was 90.0%, and the mean false alarm rate was 5.56%. For the 12 no-target filler scenes, the mean false alarm rate was 6.39%.

### Search termination

Mean overall trial duration (from scene onset to the click on the “X” to end the trial) was 18.6 s (SD = 5.90 s). Mean time spent searching (i.e., overall trial duration minus periods of report, see Fig. 2) was 12.9 s (SD = 4.89 s).

If a participant detected two targets, they could confidently end the trial, but for all other trials, participants ended the trial with the possibility that there may have been an additional, undetected target or targets. The key analysis, then, was whether participants terminated these trials earlier after having found a first target than when no first target had been found. That is, we assessed whether the SOS effect on detection rates was accompanied by an early termination effect after having found a first target.

To do this, we compared total search times for two trial types (Fig. 3B): (1) single-target, LS scenes in which the target was not detected and (2) dual-target scenes in which the HS target was detected but the LS target was not. The former provided a baseline measure of how long the participant searched before terminating the trial when a single, LS target was missed. The latter provided a measure of how long a participant searched before terminating when an HS target was detected but an LS target in the same scene was missed. For the latter measure, we subtracted from the total trial time the time taken to respond to the detected HS target (i.e., the sum of search periods A and B in Fig. 2). Early termination after having found an HS target would be characterized by shorter search time for the latter trial type than for the former. Indeed, this was the case. Mean search time for single-target trials in which the LS target was missed was 16.49 s (SD = 9.12 s). Mean search time for dual-target trials in which the HS target was detected but the LS target missed was 12.81 s (SD = 6.65 s). These reliably differed, *t*(59) = 3.91, *p* < .001, *d*_*z*_ = 0.51, indicating that participants tended to search for almost four fewer seconds when a first target was detected in a scene than when no target was detected.

### Individual differences

The above analyses demonstrate both an SOS effect on detection rates and an early termination effect after having detected a first target. We sought to determine whether these two effects were related at an individual participant level. To do this, we examined whether individual differences in termination time after detecting an HS target predicted (1) differences in LS detection rates in dual-target scenes and (2) the difference in LS detection rate as a function of single- or dual-target scene (i.e., the SOS effect).

To obtain a measure of termination time that was independent of the outcome measures to be modeled, we used single-target, HS trials on which the target was detected. For each participant, we calculated the mean time from the end of the HS report period to the end of the trial, that is, the amount of time the participant searched *after* having detected an HS target before terminating the trial (search period B in Fig. 2). We term this a *post-target search time* measure. This measure varied substantially among participants, from a minimum of 2.4 s to a maximum of 21.2 s.

As illustrated in Fig. 3C, the post-target search time measure (derived from HS, single-target trials) reliably predicted detection rates for LS targets in dual-target scenes, *r* = 0.54, *p* < .001. Participants who tended to search longer after finding an HS first target detected a higher proportion of LS targets (see also Adamo et al., 2018). To examine the SOS effect, we calculated an SOS difference score for each participant (detection rate for LS targets in single-target scenes minus detection rate for LS targets in dual-target scenes). Positive values indicate an SOS effect. As illustrated in Fig. 3D, the SOS difference score was also reliably related to the post-target search time measure, *r* = − 0.32, *p* = .014. Participants who tended to search for a shorter duration after finding an HS first target tended to have a larger SOS effect.

In sum, Experiment 1 produced an SOS effect that was modulated by inspection time. LS faces were detected at a lower rate when the same scene also contained an HS target. This effect was observed in conjunction with an early termination effect: The total time devoted to search was lower when participants found an HS target and missed the LS target in that scene than when they missed an LS target in a single-target scene. Finally, the time devoted to search after finding an HS target reliably predicted the SOS effect. Participants who were more prone to early termination tended to have higher rates of SOS. Thus, the results indicate that premature termination makes a substantial contribution to SOS in the present task.

## Experiment 2

In Experiment 2, we conducted a reduced version of Experiment 1 in a sample of radiologists. These data were collected from attendees at the 2023 European Congress of Radiology in Vienna. Given constraints on participant recruitment, we were able to collect data from only 18 participants, which falls substantially below the estimated sample size of 40 necessary to ensure sufficient power. Thus, the results should be considered as supplemental to those reported in Experiment 1.

### Method

#### Participants

Eighteen radiologists participated (five female, 12 male, one not reporting). One participant did not complete the demographics and experience questionnaire. For the remaining 17, nine were residents, and eight were board-certified radiologists. They listed experience that ranged from 3 months to 20 years (M = 6.09 years, SD = 7.25 years). The estimated number of yearly mammograms read ranged from 0 to 10,000 (M = 2,825, SD = 3,714).

#### Apparatus

Participants completed the experiment using a laptop computer provided by the experimenter.

#### Stimuli

The experimental scenes were the same as in Experiment 1.

#### Procedure

Participants first completed a practice session of five trials: one dual-target scene, three single-target scenes (two LS and one HS), and one no-target scene. Then, they completed the main session of 30 trials: 18 experimental scenes (six dual-target, six single-target HS, and six single-target LS) and 12 filler scenes (six single-target HS and six target absent). Trial order was randomly determined. The experimental scene items were counterbalanced across six groups of three participants each. Participants were instructed to complete all scenes within 15 min, and the countdown timer was modified accordingly. One participant exceeded the allowed time by four minutes. When this occurred, the participant started to see negative numbers indicating that they had exceeded the allotted time, but the experiment was not terminated. Our goal was to introduce a time pressure without necessarily compromising data acquisition, particularly the implementation of the full matched-triplet design. As in Experiment 1, feedback was provided in the practice session but not in the main session.

## Results and discussion

We first report detection rates to assess the presence and magnitude of the predicted SOS effect. We then assess search termination times to examine whether the SOS effect can be attributed to early termination after having found a first target in dual-target scenes. Note that due to a programming error, response times were not recorded for detection confidence, expression choice, and expression confidence. Thus, analyses that depended on the subtraction logic illustrated in Fig. 2, such as the individual differences analyses, were not possible. The rates and response times for all possible trial outcomes are reported in Table 2.

**Table 2:** Experiment 2 results

	Percentage of trials	Total time before detection (s)^a^		Total trial time (s)
		First target	Second target	
2 Target				
Both detect: HS first	43.52	2.99	12.10	22.32
Both detect: LS first	3.70	*	*	*
HS detect only	47.22	5.14	NA	21.40
LS detect only	2.78	*	NA	*
Both miss	2.78	NA	NA	*
1 Target HS				
Detect	96.30	3.52	NA	21.35
Miss	3.70	NA	NA	*
1 Target LS				
Detect	68.52	12.54	NA	30.35
Miss	31.48	NA	NA	31.89
0 Target filler		NA	NA	28.30

^a^Time from trial onset to target detection, including report time for any detections or false alarms that occurred earlier in the trial

*: More than 25% of participants had no observations, precluding a stable estimate of the mean

### Detection rates, detection times, and false alarm rates

**Fig. 4:**
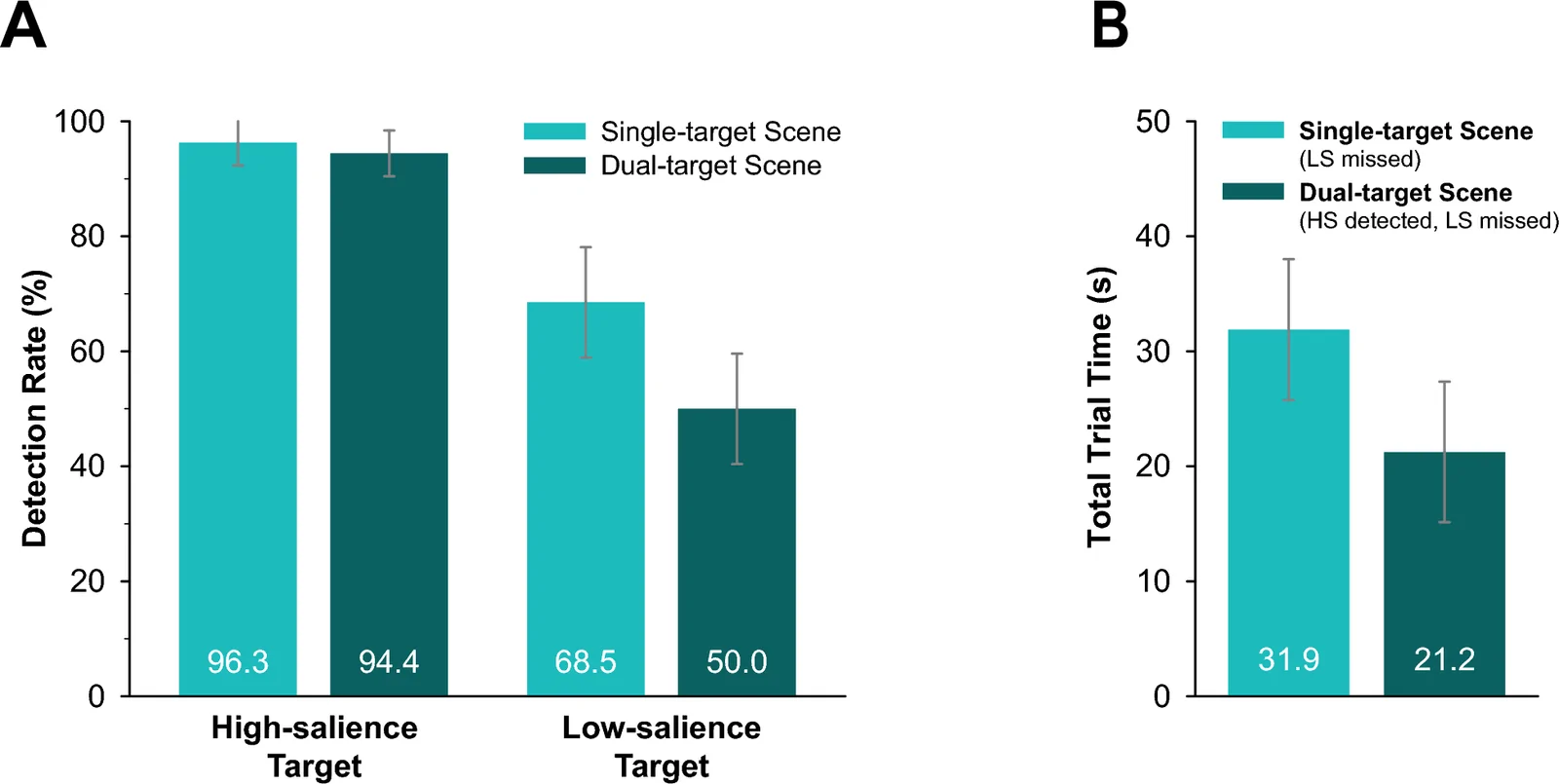
Experiment 2 results. **A** Detection rates as a function of target salience and the number of targets in a scene. Error bars are within-participant 95% confidence intervals based on the error term of each of the grouped contrasts. **B** Total trial time for (1) single-target scenes in which the LS target was missed and for (2) dual-target scenes in which the HS was detected and the LS target missed. Error bars are within-participant 95% confidence intervals

The primary analyses concerned detection rates over the experimental scenes, as illustrated in Fig. 4A. The detection rate data were entered into 2 (HS/LS face target) × 2 (single-/dual-target scene) repeated measures ANOVA. First, there was a reliable main effect of target salience, *F*(1,17) = 82.1, *p* < .001, $\eta_{p}^{2} = 0.828,$ with higher detection rate for HS targets than for LS targets. Second, there was a reliable main effect of single-/dual-target scene, *F*(1,17) = 6.61, *p* = .020, $\eta_{p}^{2} = 0.280,$ with higher detection rate for targets that appeared in single-target scenes than in dual-target scenes. Finally, there was a reliable interaction, *F*(1,17) = 8.05, *p* = .011, $\eta_{p}^{2} = 0.321.$

HS targets were detected at near-ceiling levels, with a mean detection rate of 96.3% when the HS target appeared in isolation and 94.4% when it appeared in the presence of an LS target in dual-target scenes, *t*(17) = 0.70, *p* = .495, *d*_*z*_ = 0.16. For LS targets, the detection rate was reliably higher when an LS target appeared in isolation (68.5%) than when an LS target appeared in the presence of an HS target (50.0%), *t*(17) = 2.87, *p* = .005, *d*_*z*_ = 0.68.

We also calculated SOS by considering only those dual-target trials where an HS face was the first or only target detected. The grand participant means for the percentage of trials in each of the five outcomes on dual-target trials were: (1) Both targets detected and HS detected before LS (43.5%), 2) both targets detected and LS detected before HS (3.7%), 3) HS detected and LS missed (47.2%), 4) LS detected and HS missed (2.8%), and 5) both missed (2.8%). The analysis contingent on the HS face being the first or only target detected was again limited to trial outcomes 1 and 3. Under this contingency, the mean detection rate for LS targets was 47.4%. Applying the Becker et al. correction yielded an expected contingent LS detection rate of 66.3%. The observed contingent LS detection rate in dual-target scenes (47.4%) remained significantly lower than this corrected expectation, *t*(17) = 2.78, *p* = .013, *d*_*z*_ = 0.66. Thus, the SOS effect was also observed when considering the probability of LS detection after HS detection.

Confidence in the presence of a detected target was analyzed as a function of HS/LS and single-/dual-target scene (Means: HS single = 4.90, HS dual = 5.0, LS single = 3.98, LS dual = 4.17). There was a main effect of salience, *F*(1,17) = 23.99, *p* < .001, $\eta_{p}^{2} = 0.585,$ but no effect of single-/dual-target scene, *F*(1,17) = 0.86, *p* = .367, $\eta_{p}^{2} = 0.048,$ and no interaction, *F*(1,17) = 0.081, *p* = .780, $\eta_{p}^{2} = 0.005.$

For accuracy of expression report (Means: HS single = 92.6%, HS dual = 93.3%, LS single = 64.6%, LS dual = 74.4%), there was a main effect of salience, *F*(1,17) = 25.33, *p* < .001, $\eta_{p}^{2} = 0.598,$ but no effect of single-/dual-target scene, *F*(1,17) = 0.883, *p* = .361, $\eta_{p}^{2} = 0.049,$ and no interaction, *F*(1,17) = 0.765, *p* = .394, $\eta_{p}^{2} = 0.043.$ For expression confidence (Means: HS single = 4.73, HS dual = 4.68, LS single = 3.45, LS dual = 3.46), there was a main effect of salience, *F*(1,17) = 67.86, *p* < .001, $\eta_{p}^{2} = 0.800,$ but no main effect of single-/dual-target scene, *F*(1,17) = 0.028, *p* = .870, $\eta_{p}^{2} = 0.002,$ and no interaction, *F*(1,17) = 0.055, *p* = .818, $\eta_{p}^{2} = 0.003.$

As in Experiment 1, false alarm rates were low. For the 36 experimental stimuli, participants clicked on a location that did not contain a face on an average of 3.70% of trials (SD = 5.87%) for single-target scenes and 2.78% of trials (SD = 6.39%) for dual-target scenes.

### Search termination

Mean overall trial duration (from scene onset to the click on the “X” to end the trial) was 26.9 s (SD = 4.7 s). To examine early termination, we again compared total search times for two trial types: (1) single-target, LS scenes in which the target was not detected and (2) dual-target scenes in which the HS target was detected but the LS target was not (Fig. 4B). Two participants who detected all of the LS targets in the single-target scenes were eliminated from this analysis. Mean trial time for single-target scenes in which the LS target was missed was 31.89 s (SD = 16.89 s). Mean trial time for dual-target scenes in which the HS target was detected but the LS target missed was 21.24 s (SD = 6.66 s). Note that for this latter measure, the time taken to report the HS target *was not* subtracted from the total trial time, given the absence of data for the timing of the response that ended the report period. Mean report time for HS targets in Experiment 1 was 3.81 s, so the actual time devoted to search was likely to be closer to 17.5 s, rather than 21.24 s. Nevertheless, these measures differed reliably, *t*(15) = 2.71, *p* = .016, *d*_*z*_ = 0.68, indicating that participants tended to terminate search relatively early after having detected a first target in a scene.

In summary, the results with a sample of radiologists replicated key aspects of Experiment 1, including the basic SOS effect and an early termination effect after finding a first target. These effects were numerically larger with radiologists compared with naïve participants, although the small sample of radiologists and the reduced number of trials limit comparison. It is also noteworthy that radiologists produced the same basic effects despite spending significantly longer on each image than did naïve participants (an average of 26.9 s compared with 18.6 s) and achieving higher overall detection rates. Thus, although radiologists have developed expertise in reading medical images, they have not necessarily developed strategies to avoid SOS that generalize to a non-radiological search task (see also Nodine & Kundel, 1997). One possible reason for the numerically larger SOS effect with radiologists could be due to time constraints (Samuel et al., 1995). The instruction to finish the session at an effective rate of 30 s per scene could have generated pressure to terminate inspections, especially since radiologists used almost all the allotted time, on average. Note that time pressure was less likely to have contributed substantially to the basic SOS effect among naïve participants in Experiment 1, however, since these participants generally did not come close to using all the time available to them. (On average, they completed the experiment with 12.8 s remaining on the clock.)

## General discussion

This study re-evaluated the role of premature termination as a cause of SOS errors using a novel task that incorporated the image complexity and task structure of 2D radiological image reading while at the same time providing for experimental control and the use of participants without extensive training. In addition, we restricted the time available to 30 s per image. The ghosts-in-the-forest task, as we have referred to it here, required participants to find semi-transparent faces that were integrated within backgrounds of complex forest and jungle scenes. The faces were either of relatively high salience or low salience, similar to different types of abnormalities in 2D radiological images. Unlike item-based search tasks that require the discrimination of clearly segmented, discrete stimuli, as prespecified targets or distractors, the ghosts-in-the-forest task requires the detection of unknown objects that are camouflaged within the background to varying degrees, again similar to detecting abnormalities in 2D medical images.

The results provided clear evidence that the observed SOS effect was caused, to a substantial degree, by early search termination after having found a first target, both for naïve participants (Experiment 1) and trained radiologists (Experiment 2). First, we observed a substantial SOS effect. The detection rate for LS targets was reliably lower when LS targets appeared in the same scene as an HS target compared with when identical LS targets appeared as the only target in a scene. This was observed both for overall LS detection rates and for LS detection rates contingent on prior HS target detection. Second, the SOS effect was accompanied by an early search termination effect after having found an HS target. Specifically, the overall time devoted to search was lower for (1) dual-target trials when an HS target was detected but the LS target in the same scene was missed compared with (2) single-target trials when the LS target was missed. Third, LS detection rates and SOS were related to individual differences in the time devoted to search after having detected an HS target. (This was evaluated for naïve participants but could not be evaluated for radiologists.) Specifically, *post-target search time* reliably predicted both LS detection rates and the magnitude of the SOS effect: Participants who tended to persevere in search longer after having detected an HS target tended to detect a higher percentage of LS targets in dual-target scenes and tended to have lower rates of SOS. In sum, SOS errors were associated with premature termination of image inspection, consistent with the original accounts of SOS in terms of search “satisfaction” or, more neutrally, decision-level processes concerning when to terminate image inspection (Smith, 1967; Tuddenham, 1962). Of course, extension of the present results to the causes of SOS in radiology itself will require studies using radiologists and radiological images. However, the basic design of the present task can provide a blueprint for how such studies might be implemented.

The clear support for premature termination as a substantial cause of SOS errors found in the present study stands in contrast to the broader literature, as summarized in Adamo et al. (2021). That review discussed the evidence for premature termination as a source of SOS errors as mixed at best, both in the radiology literature and in the more cognitively oriented literature on visual search. With respect to the radiology literature, studies using actual radiological images require the use of radiologists as participants, because they must know how to read the images. This limits the number of participants that can be tested as well as the kinds of stimulus control that can be implemented. Possible reasons for the mixed results in these studies, therefore, include that individual studies may have been under-powered, had idiosyncratic samples, or were not able to manipulate relevant aspects of the images or task consistently across studies. Moreover, studies in this tradition have tended to measure overall inspection time (e.g., Berbaum et al., 1991) rather than isolating the time devoted to visual search, as we did in the present study. That is, total inspection time for dual-target trials will often include the time taken to attend to, perceptually process, and potentially report the first target, overestimating the time devoted to search per se, which reduces the ability to detect an early termination effect. Our subtraction method provides a means to directly compare estimates of the time devoted to visual search across single- and dual-target trials. It is also worth emphasizing that one study in the radiology literature that did examine search time after detecting a first target (rather than total inspection time) also found an early termination effect (Berbaum et al., 2013).

With respect to mixed evidence for early termination in studies using traditional visual search tasks, future research will be required to isolate the specific task differences that generate the difference in results. However, we see several dimensions on which our task might plausibly have supported a finding of early termination after detecting a first target: (1) the use of highly camouflaged targets that were not physically segregated from a complex, contour-dense background, making it difficult to know if search had been exhaustive; (2) LS targets that were very difficult to detect; which led to (3) relatively long inspection times that were potentially taxing and could have provided an incentive to terminate search, particularly after having detected a first target; (4) the relatively low prevalence of dual-target images (Stothart & Brockmole, 2019); and (5) the pressure to terminate search given limited time to inspect all of the images.

Although the current findings implicate premature termination as a cause of SOS errors, they do not rule out additional contributions from other sources, such as those proposed by the perceptual-set and resource-depletion hypotheses. The ghosts-in-the-forest task provides an opportunity for testing additional possible causes of SOS errors. Integrating eye-tracking with the task, for example, would allow one to test whether patches of the image with similar stimulus characteristics to an initially detected face predict where else in the image observers tend to dedicate inspection time (Mello-Thoms, 2006; Mello-Thoms et al., 2014). A positive finding would provide support for a role of perceptual set in generating SOS errors. Because participants do not require extensive training to perform the ghosts-in-the-forest task, and the stimulus images can be manipulated relatively easily, the range of manipulations, including the addition of a secondary task to test for effects of resource depletion, or the introduction of additional categories of objects to test for effects of perceptual and conceptual similarity, is extensive. It seems likely that a complex combination of causes, ranging from higher order meta-cognitive effects like premature termination, to lower-level perceptual-set and resource-limitation-related causes, contribute to SOS errors. The ghosts-in-the-forest task provides a rich testbed for identifying the various sources and their interactions.

In summary, the present results provide evidence that premature termination of image inspection is a source of SOS errors. This is contrary to the general tenor of the literature, which has relegated premature termination to a relatively minor cause. This contrast may reflect differences in task and differences in power and study design. The task introduced in the current study provides a method for testing additional potential causes of SOS errors in a domain that is well matched to the real-world task of medical image reading.

## Data Availability

The study materials and the datasets analyzed during the current study are available from the corresponding author on reasonable request.
